# Radiographic evaluation of osteoarthritis of the hip: An inter-observer study of 61 hips treated for late-detected developmental hip dislocation

**DOI:** 10.3109/17453674.2012.665331

**Published:** 2012-04-24

**Authors:** Terje Terjesen, Ragnhild B Gunderson

**Affiliations:** ^1^Department of Orthopaedics; ^2^Department of Radiology, Oslo University Hospital, Oslo, Norway; Correspondence: terje.terjesen@rikshospitalet.no

## Abstract

**Background and purpose:**

Different methods have been used to classify osteoarthritis (OA) of the hip. We evaluated the reliability of different classifications in order to find which grading system is most appropriate for use in clinical practice.

**Patients and methods:**

49 patients (61 affected hips) with late-detected developmental dislocation of the hip (DDH) were studied. The mean age at follow-up was 45 (32–49) years. 3 classifications of OA were compared. The gradings by Kellgren and Lawrence (1957) (K&L) and Croft et al. (1990) are global visual assessments based on osteophytes, cysts, subchondral sclerosis, and narrowing of the joint space. The third classification is based on narrowing in the upper, weight-bearing part of the joint and defines as OA a minimum joint space width (JSW) of less than 2.0 mm at the narrowest part. 2 experienced observers, one radiologist and one orthopedic surgeon, assessed and measured the radiographs.

**Results:**

Minimum JSW (< 2.0 mm in 9 hips) gave the best inter-observer agreement (kappa value = 0.87). Using the K&L grading, inter-observer agreement was moderate (kappa = 0.55), but kappa increased when the number of categories was reduced from 5 to 3 (no OA, mild OA, and severe OA). The Croft classification gave similar agreement as the K&L grading. The intra-observer agreement was better than inter-observer agreement, irrespective of the grading system. There was a good accordance between the minimum JSW and the 2 other methods.

**Interpretation:**

Joint space narrowing using a minimum JSW of < 2.0 mm as criterion for OA was the simplest and most reproducible classification in long-term follow-up of patients with DDH. A classification based on global visual assessment can be used in addition if only hips with severe OA are included.

Osteoarthritis (OA) of the hip is the endpoint in follow-up studies of developmental dislocation of the hip (DDH) when comparing the outcome of different treatment regimes (Malvitz and Weinstein 1994, Angliss et al. 2005, Thomas et al. 2007). Different classifications of OA have been used, and our knowledge about the relationship between them is limited. Thus, comparison between long-term studies is difficult. A simple and reproducible grading system for use in future studies would be desirable.

OA of the hip can be evaluated by overall global assessment (Kellgren and Lawrence 1957, Croft et al. 1990, Tönnis and Heinecke 1999) or by measurement of the joint space width (JSW) (Croft et al. 1990, Jacobsen and Sonne-Holm 2005). Although measurement of JSW has been found to be more reproducible than gradings based on visual assessment in epidemiological studies and in certain patient groups (Croft et al. 1990, Troelsen et al. 2010), no such comparison has been performed in patients with DDH.

The aims of the present study were to answer the following questions: 1. How reproducible are different radiographic classifications of OA in long-term follow-up of patients with DDH?

2. Which classification is most suitable for use in clinical practice and research?

## Patients and methods

Patients recruited to the study had been treated for late-detected hip dislocation in our hospital between 1958 and 1962. They were included in the study if they met the following criteria: no other congenital anomalies, no neuromuscular disorders, and radiographs available for follow-up longer than 30 years. With these criteria, 49 patients (40 females) with 61 affected hips were included. The treatment principles (closed reduction after preliminary traction) have been reported previously (Terjesen and Halvorsen 2007). Mean age at reduction was 19 (4–65) months. Mean age at follow-up was 45 (44–49) years in patients who had not undergone total hip replacement (THR). In 7 patients with THR (8 hips), the last preoperative radiograph was used for the present evaluation, at a mean patient age of 40 (32–47) years.

The study was approved by the Regional Committee of Medical Research Ethics. Informed consent was obtained from all the patients.

### Radiographic evaluation

Anteroposterior radiographs of the pelvis were taken with the patient in the supine position, which is the standard procedure in our hospital. No statistically significant differences in joint space width according to patient position (supine or standing) have been reported (Troelsen et al. 2008, Terjesen and Gunderson 2011). Film-to-focus distance was 120 cm, and all radiographs included the pelvis with both hips, centered 3 cm above the pubic symphysis. The legs were parallel with the patella pointing straight upward.

One radiologist (RBG; observer 1) and one orthopedic surgeon (TT; observer 2), each of whom have more than 30 years of experience, independently evaluated and measured the radiographs. Before reading the radiographs, the observers trained together to reach consensus about landmarks to be used. The radiographs were magnified 4 times when measuring JSW, in order to obtain better visualization of the landmarks. Radiographic evaluation was performed in the 61 dysplastic hips (24 of which were bilateral) and in the 37 contralateral hips that were unaffected. None of the unaffected hips had severe OA, and these hips were therefore not included in this inter-observer study.

OA was evaluated using the overall qualitative assessments of Kellgren and Lawrence (1957) and Croft et al. (1990). Both classifications are based on the radiographic features of osteophytes on the joint margins, cystic areas, sclerosis of subchondral bone, narrowing of the joint space, and altered shape of the femoral head.

Kellgren (1963) described 4 grades of hip OA: grade 1 (doubtful OA), possible narrowing of the joint space medially and possible osteophytes around femoral head; grade 2 (mild OA), definite narrowing of the joint space inferiorly, definite osteophytes, and slight sclerosis; grade 3 (moderate OA), marked narrowing of the joint space, slight osteophytes, some sclerosis and cyst formation, and deformity of the femoral head and acetabulum; and grade 4 (severe OA), gross loss of joint space with sclerosis and cysts, marked deformity of the femoral head and acetabulum, and large osteophytes.

Croft et al. (1990) graded OA into 5 categories: grade 1, osteophytosis only; grade 2, joint space narrowing only; grade 3, two of osteophytosis, joint space narrowing, subchondral sclerosis, and cyst formation; grade 4, three of the same features as above; and grade 5, as in grade 4 but with deformity of the femoral head.

Joint space width (JSW) was measured in the upper, weight-bearing part of the joint according to Jacobsen and Sonne-Holm (2005). The shortest distance between the femoral head and acetabulum was measured at 3 locations: the lateral and medial margins of the subchondral sclerotic line (sourcil) and along the vertical line through the center of the femoral head. The minimum JSW was used for diagnosis of OA. If minimum JSW was outside the 3 standard locations, an additional measurement at the site of maximum narrowing was performed. The definition of OA is a minimum JSW of less than 2.0 mm. In order to perform an intra-observer analysis, observer 2 repeated the assessments of OA more than 3 weeks after the first assessments.

Intra- and inter-observer variation were analyzed with kappa statistics. 95% confidence intervals (CIs) were calculated for the kappa values. Kappa of 0.4–0.6 indicates moderate agreement, kappa of 0.6–0.8 means good agreement, and kappa above 0.8 means very good agreement.

## Results

The inter-observer variation according to the classification of Kellgren and Lawrence (K&L) (Table 1) showed agreement in 50 of 61 dysplastic hips. The kappa value was 0.55 (CI: 0.35–0.72; p < 0.001). When the OA grades were reduced from 4 to 2, by including grade 1 into hips with no OA, keeping grade 2 unchanged (mild OA), and combining groups 3 and 4 into one group (severe OA), the inter-observer agreement rose to 56 of 61 hips and kappa was 0.72 (CI: 0.49–0.89).

**Table 1.** Inter-observer variation using the Kellgren and Lawrence (K&L) classification (number of hips)

K&L grades	Observer 2					
	0	1	2	3	4	Total
Observer 1						
0	44	0	0	0	0	44
1	5	0	0	1	0	6
2	1	1	0	1	0	3
3	0	0	1	2	0	3
4	0	0	0	1	4	5
Total	50	1	1	5	4	61

**Figure:**
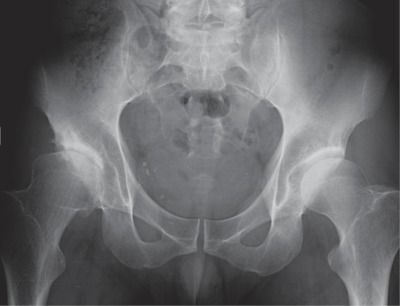
Long-term follow-up radiograph of a 44-year-old man where there was discrepancy between the observers in the assessment of the right hip. Using the minimum JSW method, observer 1 found no OA (a minimum JSW of 3.2 mm) whereas observer 2 found OA (a minimum JSW of 1.8 mm). Both observers found severe OA by Kellgren and Lawrence (grade 3) and Croft (grade 4).

Using the Croft classification (Table 2), there was agreement between the observers for 45 of the 61 hips. Kappa statistics was not possible because observer 2 had no hips of grade 2. When grade 1 was incorporated into the normal hips, when grades 2 and 3 were combined into one group (mild OA), and when groups 4 and 5 were combined (severe OA), the agreement rose to 54 of 61 hips and kappa was 0.65 (CI: 0.41–0.83).

**Table 2.** Inter-observer variation using the classification by Croft et al. (number of hips)

Croft grades	Observer 2						
	0	1	2	3	4	5	Total
Observer 1							
0	38	3	0	0	0	0	41
1	5	1	0	1	0	0	7
2	3	1	0	0	0	0	4
3	0	0	0	1	1	0	2
4	0	0	0	1	1	1	3
5	0	0	0	0	0	4	4
Total	46	5	0	3	2	5	61

With the JSW classification (Table 3), there was agreement between the observers for 59 of the 61 hips and the kappa value was 0.87 (CI: 0.64–1.0). Of 10 hips found to have OA by one or both observers, there was agreement for 8 hips but the 2 remaining hips were found to be normal by one observer and abnormal by the other (Figure).

**Table 3.** Inter-observer variation using the minimum joint space width (JSW) classification (number of hips)

JSW grades	Observer 2		
	0	1	Total
Observer 1		
0	51	1	52
1	1	8	9
Total	52	9	61

The intra-observer variation in OA of the affected hips, performed by observer 2, showed a high degree of agreement regardless of method. There was agreement for 55 of the 61 hips (kappa = 0.69) when using the K&L classification, for 52 hips with the Croft method, and for 60 of the 61 hips (kappa = 0.93) when the JSW grading was used.

The relationship between the JSW classification and the 2 other classifications (modified into 3 categories) also showed good accordance (Table 4). Observer 1 classified 9 hips as showing OA by the JSW grading and only 1 of these was judged as being normal by the simplified K&L and Croft classifications. Of the 52 hips with no OA by the JSW grading, only one hip had severe OA by K&L and Croft classifications (Figure).

**Table 4.** Relationship between the minimum JSW classifications and the other classifications (observer 1, number of dysplastic hips)

	*JSW classification*		
	No OA	OA	Total
*K&L*			
No OA	49	1	50
Mild OA	2	1	3
Severe OA	1	7	8
Total	52	9	61
*Croft*			
No OA	47	1	48
Mild OA	4	2	6
Severe OA	1	6	7
Total	52	9	61

JSW: joint space width; OA: osteoarthritis.

There was a clear association between minimum JSW and the categories of the global assessment classifications. Minimum JSW (measured by observer 1) was 3.9 mm (CI: 3.6–4.2 mm) in hips with no OA according to the K&L grading and decreased to 2.2 mm (CI: 1.7–2.9 mm) in hips with mild OA and 1.0 mm (CI: 0.3–1.8 mm) in hips with severe OA.

## Discussion

Although most previous studies have used the K&L classification, there is a lack of consensus regarding its reliability. Kellgren and Lawrence (1957) found only moderate inter-observer reproducibility, with an inter-rater correlation coefficient of 0.40. One problem with the K&L classification, pointed out by Spector and Cooper (1993), is that it involves inconsistencies in the description of radiographic features by Kellgren and Lawrence themselves and overestimates the role of the osteophyte. Kellgren (1963) specified the radiographic changes of joint space medially (grade 1) and inferiorly (grade 2). This would reflect a pattern of joint space loss similar to that in rheumatoid arthritis (Resnick 2002). Since the migration pattern of the femoral head in acetabular dysplasia and DDH is usually in the superior direction, the K&L grading appears to be less appropriate in such patients.

There have not been many previous intra- and inter-observer evaluations (using kappa statistics) of the 3 classifications used in the present study, and a comparison including the present results shows several noticeable features (Table 5). Firstly, intra-observer agreement was considerably better than inter-observer agreement irrespective of OA classification. In almost all of the studies, intra-observer agreement was good or very good with kappa over 0.70. It is the inter-observer reliability that is the most important of the two, however, especially when comparing outcomes of different treatment regimes in follow-up of DDH. Secondly, inter-observer agreement improves when the number of OA grades is reduced. When we used all 5 grades of the K&L grading (including “normal”), inter-observer agreement was moderate, but improved to “good” when a modified classification with 3 grades was used. Using only 2 categories with cutoff between grades 0–1 and 2–4, Ingvarsson et al. (2000) reported good inter-observer agreement with kappa values above 0.60. Using the same simplified K&L grading, Cerhan et al. (1995) found moderate inter-observer agreement (kappa = 0.50), indicating that K&L lacks sufficient reliability even when simplified into 2 categories.

**Table 5.** Reliability of inter- and intra-observer measurements using kappa statistics

			Kappa statistics		
Authors	Method of grading	Grades	Intra-observer kappa coefficient	Inter-observer kappa coefficient	95% CI
Cerhan et al. 1995	K&L	2 (0–1 vs. 2–4)	0.84–0.92	0.50	
Ingvarsson et al. 2000	K&L	2 (0–1 vs. 2–4)	0.76	0.60–0.65	
Present study 2011	K&L	5	0.69	0.55	0.35–0.72
Present study 2011	K&L	3 (0–1, 2, and 3–4)		0.72	0.49–0.89
Croft et al. 1990	Croft	2 (0–2 vs. 3–5)	0.49	0.41	
Croft et al. 1990	Croft	2 (0–3 vs. 4–5)	0.93	0.63	
Present study 2011	Croft	3 (0–1, 2–3, and 4–5)		0.65	0.41–0.83
Croft et al. 1990	Min. JSW	2 (cutoff 2.5 mm)	0.81	0.70	
Croft et al. 1990	Min. JSW	2 (cutoff 1.5 mm)	0.83	0.79	
Present study 2011	Min. JSW	2 (cutoff 2.0 mm)	0.93	0.87	0.64–1.0

When Croft et al. (1990) used their own visual assessment score and modified this by reducing the number of grades to 2 categories, with the cutoff between grades 0–2 and 3–5, inter-observer agreement was only moderate (Table 5). When the cutoff was between grades 0–3 and 4–5, there was good agreement, indicating that agreement improves when only hips with severe OA are defined as “disease-positive”. We found better agreement with fewer grades, which indicates that the Croft classification should be simplified if used in future studies.

The global visual assessment of Tönnis and Heinecke (1999) has been used in several previous studies of follow-up in DDH, but there has been no consensus regarding its reliability. Whereas Troelsen et al. (2010) found poor inter-observer reliability with kappa values of 0–0.39, other authors have reported moderate to good reproducibility with inter-observer kappa values of 0.59 (Clohisy et al. 2009) and 0.74 (Steppacher et al. 2008). A grading system with such conflicting reliability data can hardly be recommended for routine use. Its weakness, as with the K&L and Croft gradings, is the subjective and rather unclear dividing lines between the grades.

The K&L classification has 5 grades and the Croft has 6 grades including normal, doubtful, mild, moderate, and severe OA. A modification, reducing the number of grades, would make these classifications easier to use and would improve their reliability. Since Danielsson (1967) found that osteophytes alone are not a sign of OA, grade 1 of the K&L classification (“possible osteophytes”) and grade 1 of the Croft classification (“osteophytosis only”) could be included in the group with no OA. Grade 2 in the Croft grading “joint space narrowing only” is difficult to interpret, because there is no information about how much the joint space should be reduced and it is very rare to have a substantially reduced joint space without other signs of OA. Thus, grade 2 could be combined with grade 3 and called mild OA. The 2 highest grades, 3 and 4 by K&L and 4 and 5 by Croft, could also be combined and termed severe OA, because the distinction between them seems to be rather unimportant from a clinical point of view.

We found very good inter-observer agreement with the minimum JSW classification, with a kappa value of no less than 0.87 (Table 5). The agreement was better than with the gradings based on global visual assessment, which is in accordance with previous studies (Croft et al. 1990, Ingvarsson et al. 2000). The good agreement with the JSW grading was first reported by Croft et al. (1990) and the agreement between observers was better for superior and minimum joint space than for medial joint space. When using a cutoff for OA of 2.5 mm, inter-observer kappa was 0.70, and kappa increased to 0.79 when they used 1.5 mm as the cutoff. In an epidemiological study, Lanyon et al. (2003) found a mean minimum JSW of 3.9 mm (SD 0.5) in women and a slight decrease with age occurred (from 4.0 mm at 45 years to 3.6 mm at 80 years). If the lower limit of the normal range is defined as mean – 2 SD, this limit should be 2.8 mm. Alteration of the threshold for definition of hip OA from 2.5 mm to 2.2 mm reduced the prevalence of OA from 11% to 6% (Lanyon et al. 2003). A cutoff value of 2.5 mm for OA has been used by some authors (Ingvarsson et al. 2000, McWilliams et al. 2010). However, in order to avoid a falsely high prevalence of OA, we recommend a cutoff for minimum JSW of 2.0 mm in accordance with Jacobsen and Sonne-Holm (2005) and Troelsen et al. (2010).

JSW measurements had good accordance with gradings based on global visual assessment. Thus, comparison of long-term studies of DDH patients could be sufficiently reliable, even if different OA classifications have been used. This indicates that the K&L and Croft gradings could still be used, but the categories should be as few as possible and should not exceed 3: no OA, mild OA, and severe OA. However, since the JSW classification is the most reproducible and also the simplest and fastest, this appears to be the preferred method for future studies, especially in DDH where the most important location of the joint is the upper weight-bearing part. Moreover, minimum JSW had a closer association with pain than the K&L and Croft classifications (Jacobsen et al. 2004). Digital measurements directly on the screen with 4 times enlargement of the radiograph adds to the convenience of the JSW method. Digital measurements have been found to be more accurate than traditional manual measurements on hard copies of radiographs (Conrozier et al. 1997).

Even if a hip has no OA by the minimum JSW method, global visual assessment can show severe OA (K&L grades 3–4 and Croft grades 4–5). Although this rarely occurs, such hips should probably be added to those classifed as OA by the minimum JSW method, giving the true total frequency of OA.

In conclusion, the minimum JSW method is the simplest and most reliable classification when grading the presence of hip OA in long-term studies of patients with DDH. A classification based on global visual assessment can be used in addition if only the severe grades of OA are included in the abnormal hips.
